# A comparison about the depression, anxiety, and despair levels of the parents’ of healthy children and children with Spina bifida disease

**DOI:** 10.1192/j.eurpsy.2023.1228

**Published:** 2023-07-19

**Authors:** P. Ulual, V. Ozer, G. Özpınar, I. Alataş, R. Çetiner, O. Güçlü

**Affiliations:** 1 Istanbul Basaksehir Cam ve Sakura Sehir Hastanesi; 2 private office; 3 İstanbul Bilim Üniversitesi; 4 İstanbul Eğitim ve Araştırma Hastanesi, İstanbul, Türkiye

## Abstract

**Introduction:**

Spina Bifida(SB) is a congenital disease can cause multi system disfunction and psychiatric symptoms.It is stated that the emotional and physical burden of the caregivers leads to increased levels of anxiety and depression.

**Objectives:**

According to Gargiulo’s stage model, is composed of three stages. In the first phase parents experience shock, denial and depression. During the secondary phase ambivalance, anger, and guilt is prominent, followed by the tertiary phase in which bargaining, acceptance and adjustment are observed. Adjustment is the process of reorganisation and realignment of the family needs and objectives and variates depending on the character of the family members.

**Methods:**

Beck Depression Inventory (BDI), Beck Anxiety Inventory (BAI), Beck Hopelessness Scale (BHS) were used in this research. 66 volunteering parents were included, 34 of them having child with SB and 32 of them having a healthy (non-SB) child.

**Results:**

Depression, anxiety and hopelessness scores were significantly higher in the SB group than the non-SB group. There is a significant positive correlation between education and income levels.

**Conclusions:**

It is determined that the majority of the SB group did not have any support. The greatest burden the parents have is emotional breakdown, followed by economic hardships and physical fatigue. Depression, anxiety and hopelessness scores and age of affected child are negatively correlated. The younger child’s age, the higher the scores. There is a strong, negative and opposite correlation between the parents’ ages and depression scores. Although it is almost impossible for the families to avoid from the hard and long treatment process of the clarify the reasons behind the disease, activate the protective medical services and if possible do prenatal treatment in order to lessen the postnatal degree of the disease are highly significant. Therefore, financial and moral burden of the society are reduced.

**Disclosure of Interest:**

None Declared

